# Is Mixtures’ Additivity Supported by Empirical Data? A Case Study of Developmental Toxicity of PFOS and 6:2 FTS in Wildtype Zebrafish Embryos

**DOI:** 10.3390/toxics10080418

**Published:** 2022-07-25

**Authors:** Megan E. Fey, Philip E. Goodrum, N. Roxanna Razavi, Christopher M. Whipps, Sujan Fernando, Janet K. Anderson

**Affiliations:** 1 College of Environmental Science and Forestry, State University of New York, Syracuse, NY 13210, USA; mfey01@esf.edu (M.E.F.); razavi@esf.edu (N.R.R.); cwhipps@esf.edu (C.M.W.); 2 GSI Environmental Inc., Austin, TX 78759, USA; jkanderson@gsi-net.com; 3 Center for Air and Aquatic Resources Engineering and Science (CAARES), Clarkson University, Potsdam, NY 13699, USA; sfernand@clarkson.edu

**Keywords:** PFAS, mixtures, developmental toxicity, aqueous film-forming foam

## Abstract

Per- and polyfluoroalkyl substances (PFASs) are a major priority for many federal and state regulatory agencies charged with monitoring levels of emerging contaminants in environmental media and setting health-protective benchmarks to guide risk assessments. While screening levels and toxicity reference values have been developed for numerous individual PFAS compounds, there remain important data gaps regarding the mode of action for toxicity of PFAS mixtures. The present study aims to contribute whole-mixture toxicity data and advance the methods for evaluating mixtures of two key components of aqueous film-forming foams: perfluorooctanesulfonic acid (PFOS), and 6:2 fluorotelomer sulfonic acid (6:2 FTS). Wildtype (AB) zebrafish embryos were exposed to PFOS and 6:2 FTS, both as individual components and as binary mixtures, from 2 to 122 h post-fertilization. Five treatment levels were selected to encompass environmentally relevant exposure levels. Experimental endpoints consisted of mortality, hatching, and developmental endpoints, including swim bladder inflation, yolk sac area, and larval body length. Results from dose–response analysis indicate that the assumption of additivity using conventional points of departure (e.g., NOAEL, LOAEL) is not supported for critical effect endpoints with these PFAS mixtures, and that the interactions vary as a function of the dose range. Alternative methods for quantifying relative potency are proposed, and recommendations for additional investigations are provided to further advance assessments of the toxicity of PFAS mixtures to aquatic organisms.

## 1. Introduction

Per- and polyfluoroalkyl substances (PFASs) are a large and diverse group of chemicals with wide-ranging physical, chemical, and toxicological properties. As with many emerging contaminants for which regulatory standards continue to rapidly evolve, our ability to detect greater numbers of PFASs at ever-lower concentrations continues to outpace our understanding of their dose–response relationships—particularly under conditions of chronic exposure to mixtures at environmentally relevant levels. Comprehensive conceptual frameworks for the risk assessment of mixtures have been developed to help guide regulatory policy decisions and risk management of mixtures [1,2,3,4]. However, key data gaps remain in determining the most scientifically defensible path forward for many PFAS mixtures. The result is a patchwork of screening levels and advisories for PFASs, many of which reflect simplifying assumptions regarding concentration and dose additivity [5]. As of the writing of this manuscript, the U.S. Environmental Protection Agency (U.S. EPA) has released draft Aquatic Life Ambient Water Quality Criteria for perfluorooctanesulfonic acid (PFOS) and perfluorooctanoic acid (PFOA) for public comment [6,7]. The proposed chronic water column concentration protective of aquatic life in fresh waters is 8.4 µg/L for PFOS and 94 µg/L for PFOA. The U.S. EPA acknowledges that complex mixtures of PFASs are present in the surface waters of the U.S., and briefly summarizes candidate mixture methods that may be applied, but stops short of proposing an approach, stating [6] (p. 48) “… the ecological effects of these potential PFAS mixtures are poorly understood”.

The present study aims to contribute whole-mixture toxicity data and advance the methods for evaluating such data for two key components of aqueous film-forming foams (AFFFs): PFOS, and 6:2 fluorotelomer sulfonic acid (6:2 FTS). Wildtype (AB) zebrafish embryos were selected as the animal model because of their suitability for evaluating multiple effect endpoints relevant to fish and humans. For example, chemically induced impairment of the swim bladder may affect not only buoyancy, but also gas exchange at the air–mucous interface of fish. This is potentially translatable to human research, because the swim bladder and mammalian lung are homologous structures [8].

A series of component and binary mixture experiments were conducted to build upon previous studies with zebrafish exposed to PFOS, 6:2 FTS, and various binary mixtures with these compounds. While mortality rates were monitored, the goal was to understand whether dose additivity is supported for sublethal exposure levels. Dose ranges for this study overlapped with the LC_50_s reported in the literature for zebrafish embryos exposed to PFOS (i.e., 2000 to 68,000 μg/L) and rainbow trout embryos exposed to 6:2 FTS (i.e., >8700 μg/L) [9,10,11,12,13,14]. The LOAELs for developmental effects (i.e., swim bladder area (SBA), body length (BL), yolk sac area (YSA)) reported in the literature for zebrafish generally range between 1000 and 8000 μg/L [10,11,15,16,17,18].

A variety of common points of departure (PODs) derived from toxicity study data are illustrated in this study, including a no-observed-adverse-effect level (NOAEL), a lowest-observed-adverse-effect level (LOAEL), a benchmark dose (BMD), and a benchmark dose lower bound (BMDL). This analysis builds upon the tiered methods for risk assessment of mixtures previously reviewed in detail [5]. Specifically, a relative potency factor (RPF) approach is evaluated using ratios of PODs for PFOS and 6:2 FTS. A key assumption in the use of RPF is that the ratio of PODs is constant, such that the assumption of additivity holds across the relevant dose ranges of the mixtures. This study applies multiple approaches to examine potential interactions between component chemicals across dose intervals applied in a binary mixture experiment, including isoboles—a graphical tool recommended by the U.S. EPA [3]. In addition, the use of full dose–response curves, rather than ratios of discrete PODs, is explored as an alternative approach to interpreting relative potency from mixture studies, relaxing the assumption of a constant RPF.

## 2. Materials and Methods

### 2.1. Test Material Sources

Perfluorooctanesulfonic acid potassium salt (PFOS, CF3(CF2)7SO3K, CAS #2795-39-3) was purchased from Sigma-Aldrich (St. Louis, MO, USA). 1H,1H,2H,2H-perfluorooctanesulfonic acid (6:2 FTS, C8H5F13O3S, CAS #27619-97-2) was purchased from SynQuest Laboratories (Alachua, FL, USA). ACS-grade dimethyl sulfoxide (DMSO, (CH3)_2_SO, CAS #67-68-5) was purchased from VWR (Radnor, PA, USA). ACS-grade potassium chloride (KCl, CAS #7447-40-7) and ACS-grade monopotassium phosphate (KH_2_PO_4_, CAS #7778-77-0) were purchased from Sigma (St. Louis, MO, USA). ACS-grade sodium chloride (NaCl, CAS #7647-14-5) and ACS-grade magnesium sulfate (MgSO_4_, CAS #7487-88-9) were purchased from Thermo Fisher Scientific (Waltham, MA, USA). ACS-grade calcium chloride (CaCl_2_, CAS #10043-52-4) and ACS-grade disodium phosphate (Na_2_HPO_4_, CAS #7558-79-4) were purchased from Baker Chemical Company (Phillipsburg, NJ, USA). ACS-grade sodium bicarbonate (NaHCO_3_, CAS #144-55-8) was purchased from EM Industries, Inc. (Gibbstown, NJ, USA). The PFAS-free water was from a Milli-Q Q-Pod Ultrapure Water Remote Dispenser (Darmstadt, Germany), and was confirmed to be PFAS-free via mass spectrometry. PFAS analytical standards were purchased from Wellington Laboratories (Guelph, ON, Canada).

The 100 mm polypropylene Petri dishes (CAS #S29423), stainless steel fine-tip probes (CAS #17-467-615), stainless steel tweezers (CAS #17-467-136), stainless steel lab spoon (CAS #14-375-20), and aluminum weighing dishes (CAS #08-732-101) were purchased from Fischer Scientific (Hampton, NH, USA). The 1000 μL wide-bore pipette tips (CAS #3184-76) were purchased from Weber Scientific (Hamilton, NJ, USA). The glassware, bottles, probes, spoon, and Petri dishes were rinsed five times with methanol and then twice with PFAS-free water to minimize the potential for cross-contamination.

### 2.2. Dose Regimens

Dose regimens for each of three experiments consisted of a control group (T0) and five treatment groups (T1 to T5), with T1 being the lowest concentration and T5 being the highest. Each experiment was repeated three times, producing three replicates of results per experiment. Three replicates of five treatment groups were run for PFOS (nominal concentrations ranging from 0.10 to 1980 μg/L), 6:2 FTS (1 to 19,800 μg/L), and binary mixtures at 1:10 concentration ratios of PFOS to 6:2 FTS, with the same concentration ranges.

All treatment solutions were prepared via serial dilution from the stock chemical in embryonic media (E2) and dimethyl sulfoxide (DMSO), in which the concentration of DMSO was kept constant at 1% (*v*/*v*) [19,20]. All stocks, treatments, and environmental controls were analyzed by mass spectrometry before exposing the larval fish, to ensure purity and consistency with the target dosing regimen. Controls across experiments contained only E2 with 1% (*v*/*v*) DMSO. Although DMSO is commonly used as a solvent, future studies would benefit from including an additional negative control group with DMSO alone. The E2 was composed of 7.5 mM NaCl, 0.25 mM KCl, 0.5 mM MgSO_4_, 75 μM KH_2_PO_4_, 25 μM Na_2_HPO_4_, 0.5 mM CaCl_2_, and 0.35 mM NaHCO_3_ [21].

The PFOS stock solution was prepared with only DMSO. The 6:2 FTS and binary mixture stock solutions were prepared with E2 rather than DMSO, because the DMSO solution would have exceeded the 1% (*v*/*v*) baseline. Treatment groups were assigned using a 1:10 target serial dilution of PFOS to 6:2 FTS, which approximates the ratio of LC_50_s for zebrafish [22], and is within the range of POD ratios reported for rainbow trout, amphibians, and aquatic invertebrates (see Table S1).

### 2.3. Quality Assurance/Quality Control (QA/QC)

Analysis of target PFAS samples and environmental controls was performed using Thermo Vanquish LC coupled with a Thermo Altis Triple-Quadrupole Mass Spectrometer (Thermo Scientific, Waltham, MA, USA). All samples were diluted so that they contained at least 50% PFAS-free methanol. For example, the water (used in E2, rinses, and egg collection) was diluted to 50% water and 50% methanol, while the methanol used in cleaning was spiked with 50% PFAS-free water. The stock and treatment solutions were diluted with a 75% methanol–water solution between 100,000- and 10-fold, such that the concentration was within the calibration range (0.020–0.010 μg/L). All samples were diluted in 2 mL glass amber autosampler vials, and subsequently spiked with the mass-labeled PFAS to serve as internal standards for quantification.

### 2.4. Zebrafish Husbandry

Adult zebrafish (*Danio rerio*) were a second generation of AB line fish originally purchased as embryos from the Zebrafish International Resource Center (ZIRC; Eugene, OR, USA). The fish were housed in a flow-through Aquaneering (San Diego, CA, USA) stand-alone system at SUNY ESF’s Center for Integrated Research and Teaching in Aquatic Science. Housing and maintenance of the adult zebrafish were in accordance with SUNY ESF IACUC protocols. Fish were housed in 2.8 L tanks by age, and separated by sex prior to breeding, with 10–20 fish per tank. Fish were fed twice daily during the week and once per day on the weekends with Gemma 300 (Skretting, Tooele, UT, USA). Water quality was monitored daily to maintain relatively constant ranges for pH (7 to 8), conductivity (600 to 1000 μS), and temperature (28 ± 2 °C). Additional parameters, monitored monthly, were reported as follows for these experiments: 12.1 dKH alkalinity, 12 ppm O_2_, 2.5 ppm nitrate, 0.5 ppm ammonia, and 0 ppm nitrite. The fish were kept on a 14:10 h light:dark photoperiod. The water flow to each tank was adjusted so that 100% of the tank water would be replaced daily.

Zebrafish embryos were obtained by breeding adult AB zebrafish in a 1.7 L Techniplast Slope Breeding Tank (Exton, PA, USA) the night prior to embryo collection, and pulling the divider approximately 30 min before turning on the lights to induce spawning. At 2 h post-fertilization (hpf), embryos were collected in a beaker of PFAS-free water and dispensed randomly to different treatment groups. Eggs were chosen based on the presence of a furrow signaling that they were developing normally [23]. Each Petri dish represented a replicate of an experiment. Twenty translucent (developing) embryos were dispensed into each Petri dish. There were three replicates per treatment, for a total of 18 Petri dishes (5 treatments plus control × 3) and 360 fish (18 × 20) per experiment.

Embryos were monitored daily using a Nikon Model C-LEDS stereo zoom microscope (Spectra Services, Ontario, NY, USA) around the same time that the treatment solutions were added (at approximately 26, 50, 74, 98, and 122 hpf) to record mortalities and numbers of hatchlings, and to note developmental stages. Mortality was identified as the egg turning opaque/coagulating from lack of development, missing heartbeat, and failure to develop somites [24]. Dead embryos were removed from the Petri dish and discarded. Chorions of hatched embryos were also discarded. After the embryos were monitored for 5 days post-fertilization (dpf), the plates were put in a 5 °C fridge for a few hours. Euthanasia was confirmed by the lack of heartbeat under the microscope while still in the treatment solution before being plated for imaging.

### 2.5. Microscopy and Image Analysis

**Figure 1:**
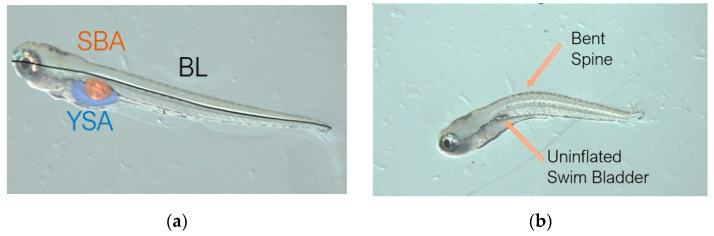
Examples of morphometric measurements taken from 5 dpf; BL = body length, SBA = swim bladder area; YSA = yolk sac length. (**a**) Example from control group showing normal development. (**b**) Example from binary mixture T5 (see Table 1 for measured concentrations of PFOS and 6:2 FTS), showing differences in SBA and BL.

Euthanized fish were oriented laterally on a 2% methylcellulose solution to be photographed on a Nikon SMZ800 stereomicroscope with an Excelis camera. Morphological measurements were carried out using the open-source software ImageJ (National Institute of Mental Health, Bethesda, MD, USA). Data collected from the images included body length (BL), swim bladder area (SBA), and yolk sac area (YSA). Measurements were modeled after the works of Parichy [25] and Martínez [10] (Figure 1a). Each measurement for each fish was performed three times, and the results were averaged for the final data point. Figure 1 shows an example of control group (Figure 1a) and treatment group (Figure 1b) images, with apparent adverse effects on BL, SBA, and YSA.

### 2.6. Statistical Analysis

Statistical analysis was performed using R version 4.2.0 (R Development Core Team, Vienna, Austria) [26]. Five effect endpoints were included in this study: mortality rate, hatch rate, BL, SBA, and YSA. Linear models were fitted to the data after checking the model assumptions with residual plots. One-way analysis of variance (ANOVA) within the emmeans (estimated marginal means) R package was used to perform multiple pairwise comparisons (significance level *p* < 0.05, Tukey’s correction) of replicate means (control vs. treatment) for each of the five endpoints within each experiment (PFOS, 6:2 FTS, and binary mixture) [27,28].

Dose–response analysis was conducted with the U.S. EPA’s Benchmark Dose Software (BMDS version 3.1.1) [4], applied to the results pooled across three replicates. Benchmark response (BMR) was defined as one control standard deviation for continuous variables (BL, SBA, and YSA). A shift in the mean response of one standard deviation equates to a 10% increase in the number of animals reaching the abnormal response level, assuming exposure results in a normal distribution of response and that there is a 1% response in the control group [29,30]. Standard PODs applied in regulatory toxicology were determined, including a no-observed-adverse-effect level (NOAEL), lowest-observed-adverse-effect level (LOAEL), benchmark dose (BMD), and 95% lower confidence limit for the benchmark dose (BMDL). In addition, exploratory data analysis was conducted to identify findings that yielded monotonic dose–response relationships and reasonable candidate dose–response models.

Whereas ANOVA supports the determination of two PODs in toxicity studies (NOAEL and LOAEL), dose–response analysis supports alternative expressions for the POD that are more easily associated with a specified response level and, therefore, are currently preferred for purposes of risk assessment [31,32]. For example, a 20% response level may describe a 20% effect concentration (EC_20_) in the context of aquatic ecological screening levels. Similarly, a 10% benchmark dose (BMD_10_) and 95% lower bound for the 10% benchmark dose (BMDL_10_) can define PODs applied for the derivation of an oral reference dose for human health risk assessment. The choice of metric used to define a POD can have important implications for how relative potency is interpreted. Furthermore, the decision to reduce toxicology study data to a POD, rather than to use all of the information from the full dose–response curve, imposes constraints that have further implications for the risk assessment of mixtures.

## 3. Results and Discussion

Table 1 summarizes the nominal and measured concentrations by treatment group. For the component experiment with 6:2 FTS, a concentration of 1.9 μg/L was detected in the control group, potentially reflecting residual chemical remaining in the mass spectrometer—given that the samples were analyzed in sequence from highest to lowest treatment, followed by the control group sample. The T4 and T5 concentrations in the PFOS component study were much higher than expected and can potentially be explained by the ubiquitous nature of PFOS in the environment. For the binary mixture experiment, the nominal concentration ratio was 1:10 (PFOS to 6:2 FTS), whereas the measured concentration ratios ranged from 1:10 to 1:33 (see Table S2).

While the measured concentrations for 6:2 FTS were very comparable (i.e., within ±10%) between the component study and the binary mixtures study, the concentrations for PFOS were 2–10-fold higher in the component study across the treatment groups. Two factors contribute to the study-specific differences in exposure concentrations for PFOS: for the component study, the measured concentrations were 2–4-fold higher than the nominal concentrations, whereas for the binary mixtures study, the measured concentrations were consistently 2–3-fold lower than the nominal concentrations. It is unclear why this pattern was observed for PFOS but not for 6:2 FTS. This observation suggests that the chemical mixture may influence the mechanisms of mass loss of PFOS during the experiment, which may warrant closer examination of comparable binary mixture studies reported in the literature.

### 3.1. Ratios of NOAELs and LOAELs

Statistically significant (*p* < 0.05) differences in mean responses were observed between controls and treatment groups for selected effect endpoints. The results of ANOVA and dose–response analysis are summarized below. Supplementary Tables S3 and S4 provide more detailed intra-experiment results, including individual fish measurements for each of three replicates per experiment.

The five effect endpoints examined were evaluated by ANOVA (Table 2). Only PFOS and the binary mixture experiments had statistically significant results, so 6:2 FTS was omitted from the table (see Tables S3 and S4); as such, T5 for each endpoint of 6:2 FTS was considered an unbounded NOAEL. The LOAELs for PFOS for BL were comparable for the component experiment (T4 = 2070 μg/L) and the mixtures experiment (T5 = 1570 μg/L). Likewise, the mortality rate and hatch rate endpoints were no different between the component and mixture studies. The BL and YSA endpoints demonstrated a non-monotonic dose–response relationship for exposure to PFOS, given that the response was statistically significant at T4 (2070 μg/L) but not at T5 (7480 μg/L). Flynn et al. [32] reported similar non-monotonic dose–response relationships for morphological changes in amphibians (*R. ripiens*, *A. americanus*, and *A. tigrinum*) following exposure to PFOS at concentrations ranging from 10 to 1000 μg/L.

For SBA, results of the binary mixture experiments suggested that a NOAEL occurred at T4, whereas the component PFOS study yielded an unbounded LOAEL at T1. This appears to indicate that there is an interaction between PFOS and 6:2 FTS that is protective (e.g., antagonism). A more thorough evaluation of the dose–response relationships suggests that the interaction shifts across the concentration ranges examined in the binary mixtures study, as discussed in Section 3.4.

**Figure 2:**
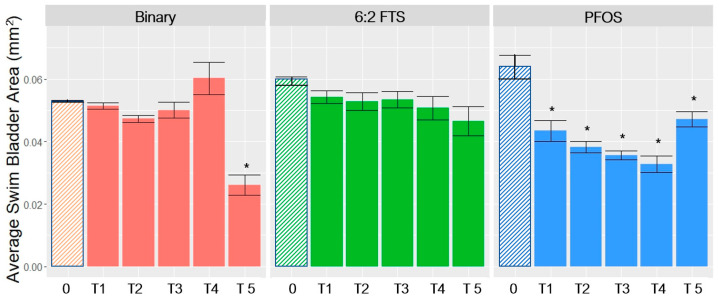
ANOVA and multiple comparison results for mean swim bladder area (SBA). * = statistically significant (*p* < 0.05). Mean ± SEM of three replicates for each treatment group. 0 = control group; T1 to T5 are treatment groups (see Table 1 and Table S1 for measured concentrations).

The mean SBA for PFOS-exposed zebrafish was statistically lower than in unexposed controls (Figure 2). This was also true for the highest dose of the binary mixture. While the differences in means (compared to the control group) for 6:2 FTS were not statistically significant, there was a trend of decreasing SBA with increasing dose.

Table 3 combines the results from Table 1 and Table 2 to show the numerical values of the NOAELs and LOAELs. For component studies, one option for quantifying relative potency is to calculate the ratio of the PODs [3,5]. If PFOS is used as the index chemical, the relative potency of 6:2 FTS is given by the ratio of the POD for PFOS divided by that of 6:2 FTS. Table 3 shows both the ratio and the inverse of this ratio for convenience, to demonstrate the factor difference more clearly in the PODs. For example, based on ratios of the unbounded PODs, the difference in relative potency between 6:2 FTS and PFOS is at least 20,000-fold for the SBA and YSA endpoints. For BL, the difference is at least 300-fold based on the ratio of NOAELs, and 7.5-fold based on the ratio of LOAELs.

### 3.2. Dose–Response Analysis—Ratio of BMDLs

Dose–response analysis was conducted for SBA for the component studies (PFOS, 6:2 FTS) as well as the binary mixtures. The other endpoints did not display a sufficiently monotonic dose–response relationship to apply the dose–response analysis methodology.

The SBA datasets were evaluated using BMDS Version 3.1.1 (U.S. EPA, Washington, DC, USA) with a benchmark response (BMR) of one control standard deviation, corresponding to a shift in the mean SBA of approximately 20% for these datasets. All candidate dose–response (D–R) curves were considered, including linear, exponential, polynomial, Hill, and power curves. Although there were five treatment groups—which is typically sufficient to establish a D–R relationship if the dose range encompasses the LOAEL—the change in SBA in the PFOS experiment exhibited a flat response for the first four treatment groups, followed by a notable change (i.e., increase in mean SBA) for the highest treatment group. This non-monotonic pattern precludes fitting D–R models, so the highest dose group (T5) was excluded from the analysis.

**Figure 3:**
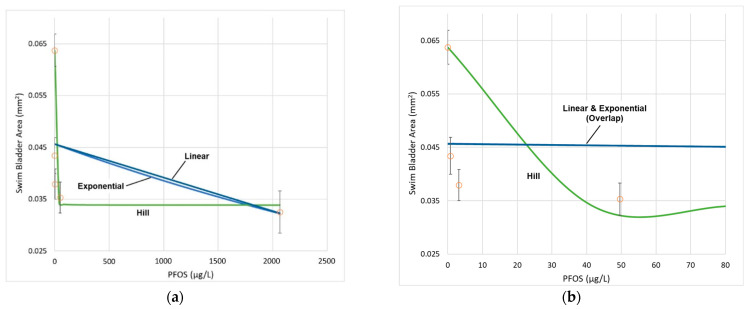

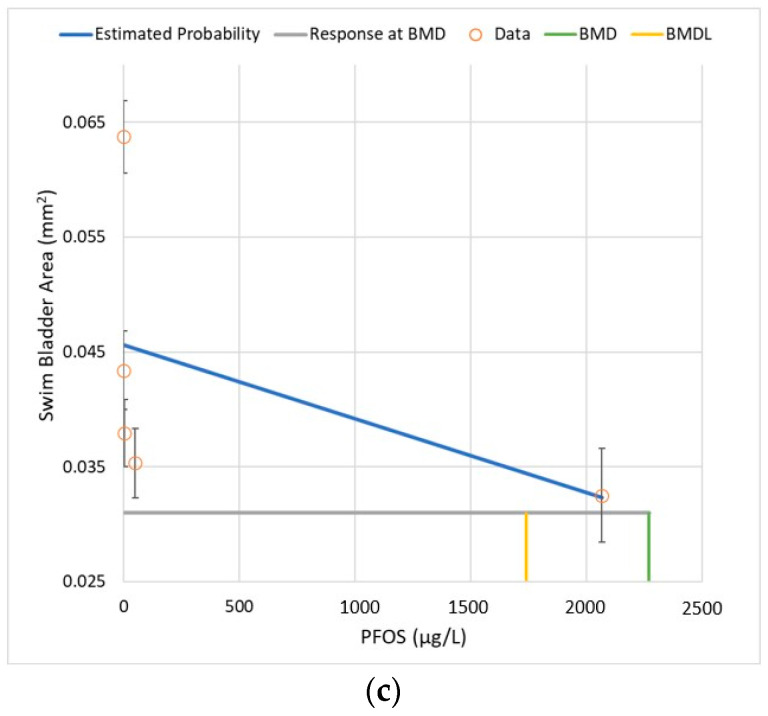
Dose–response curves for PFOS (alone) and SBA (all three replicates combined): (**a**) The uncertainty in the shape of the curve between T3 (50 μg/L) and T4 (2066 μg/L). (**b**) The same as (**a**), but for the dose range < 80 µg/L. Each model yields comparable fit statistics using BMDS 3.1.1, as well as estimates of BMD and BMDL (see Table 4) corresponding to a BMR at one control SD. (**c**) Linear D–R model showing the BMD and BMDL for BMR = control + 1 SD.

For PFOS, the notable 40-fold dose spacing gap between T3 (~50 µg/L) and T4 (~2000 µg/L) introduces uncertainty in the shape of the D–R curve within this range of exposure. Figure 3 illustrates the difference in the shapes of the mathematical D–R functions that each provide approximately the same level of precision in the fit to the PFOS dataset. The Hill model has a much steeper slope at the low-dose region (i.e., <50 µg/L), whereas the slope is steeper for the linear and exponential models at higher doses.

Despite the wide range of shapes of the D–R curves (Figure 3a), the BMDLs for PFOS vary within a narrow range (i.e., 6% relative percent difference), with the exception of the Hill model, which yields a result that extrapolates well below the lowest dose group (Table 4).

Using the linear model, the PFOS BMDL for SBA for this study—approximately 1700 μg/L—corresponds to the 20th percentile of the various PODs for toxicity studies with zebrafish reported in the literature (see Table S6 and Figure S1). The BMDL is the 95% lower confidence limit for the benchmark response at one control group standard deviation and is most analogous to a NOAEL POD. By contrast, the LOAEL of 0.76 μg/L for both SBA and YSA is unbounded for this study and is several orders of magnitude lower than the LOAELs reported in the literature. This discrepancy highlights the uncertainty associated with use of NOAEL/LOAEL PODs for evaluating relative potency.

**Figure 4:**
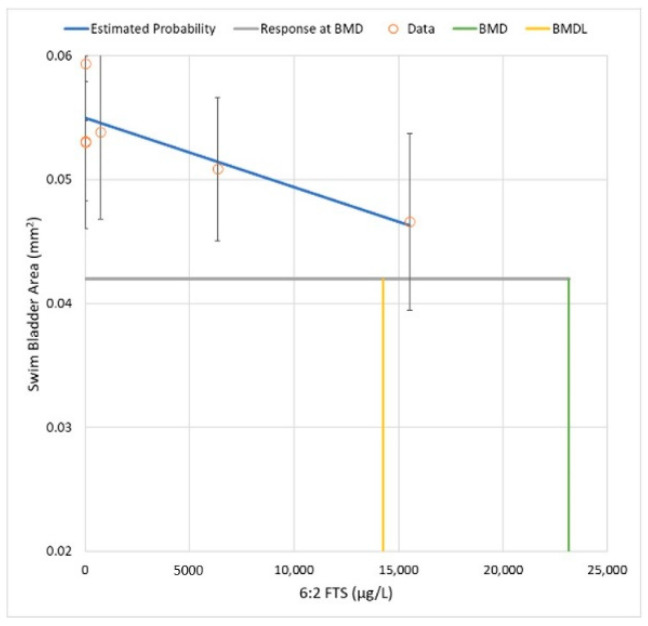
Linear D–R model’s fit to the data for 6:2 FTS (alone) and SBA (all three replicates combined). The BMD (23,200 μg/L) and BMDL (14,260 μg/L) correspond to a BMR of one control SD.

Applying a similar analysis to 6:2 FTS for the SBA effect endpoint, the D–R relationship is reasonably approximated by a linear model. Figure 4 shows the linear model’s fit to the dataset and the corresponding BMD and BMDL estimates. Compared with PFOS, the shape of the D–R curve for 6:2 FTS has greater uncertainty, given that the response at the highest dose (i.e., 22% change in mean SBA) tested in this experiment is not statistically different from the control (i.e., *p* = 0.1; T5 is an unbounded NOAEL) (see Table S5).

The ratio of the BMDLs for 6:2 FTS and PFOS is approximately 10 (i.e., 14,260 μg/L divided by 1739 μg/L = 8.2). Therefore, the ratio of BMDLs yields an estimate of relative potency that is different from the NOAEL/LOAEL approach by more than three orders of magnitude (i.e., >1:10,000 vs. 1:10).

### 3.3. Dose–Response Analysis—Full Dose–Response with Individual Chemical Experiments

Using the D–R curves for PFOS (Figure 3b) and 6:2 FTS (Figure 4), the relative doses for both components can be estimated across the full range of responses (e.g., 5% to 95%), rather than a single point on the curve, such as the BMR. The dose that corresponds to a specified response level can be readily calculated by rearranging the familiar linear equation to solve for dose. Equation (1) provides the general equation for the linear D–R model:

*R_i_* = *a* + *b* (*D_i_*) (1)

where

*R_i_* = response at the *i*th dose (e.g., SBA in units mm^2^);
*D_i_* = dose (μg/L);
*a* = intercept (mm^2^);
*b* = slope (mm^2^ per μg/L).

Equation (2) provides the general equation for calculating the percentage response as a function of the difference from the control group response:

(2) $$\begin{array}{c} \%\;response=\left[\frac{R_{0}-R_{i}}{R_{0}}\right]\times 100\%\\ R_{i}=R_{0}\left(1-\%\;response\right) \end{array}$$

where

*R_i_* = response (mm^2^) at the *i*th dose;
*R*_0_ = control group response (mm^2^) at *D* = 0 μg/L.

Substituting Equation (2) into Equation (1), and rearranging to solve for *D_i_*, yields the solution for *D_i_* at each % response level for the linear model defined by the parameters *a* and *b*.

(3) $$D_{i}=\frac{R_{0}\left(1-\%\;response\right)-a}{b}$$

**Figure 5:**
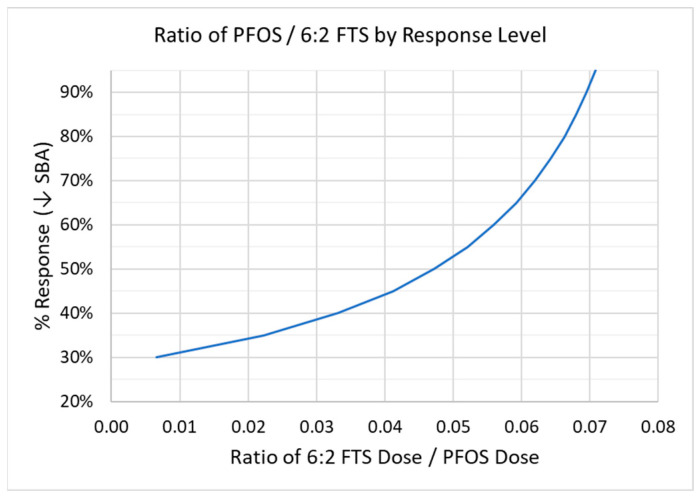
Variability in relative potency (given by the ratio of 6:2 FTS divided by PFOS) by response level, defined as the % reduction in mean SBA. Based on linear D–R models fitted to separate component experiments with PFOS and 6:2 FTS.

Applying Equation (3) to the results of the component experiments, Table 5 and Figure 5 show how the ratio of 6:2 FTS to PFOS varies across a relevant range of % responses, noting that the intercept of the PFOS results begins at an approximate 27% reduction in mean SBA. The relative potency (given by this ratio) varies by approximately a factor of 7 (0.01 to 0.07) for the range of 30% to 95% reduction in mean SBA. The corresponding inverse of this ratio (i.e., PFOS to 6:2 FTS) is approximately 14 to 150.

### 3.4. Dose–Response Analysis—Full Dose–Response with Binary Mixtures

Equation (3) can also be applied to the binary mixtures experiment to determine whether the results are similar to those of the component experiments. An inherent assumption is that the response is a function of the potency-weighted sum of doses, and that any interactions would be reflected in the observed shift in the shape (or slope) of the D–R curve. Similar to the analysis of individual experimental D–R functions, by applying the full D–R functions rather than a single POD, the relative contributions of both chemicals of the mixture can be examined across a broader range of potential environmental exposure conditions.

**Figure 6:**
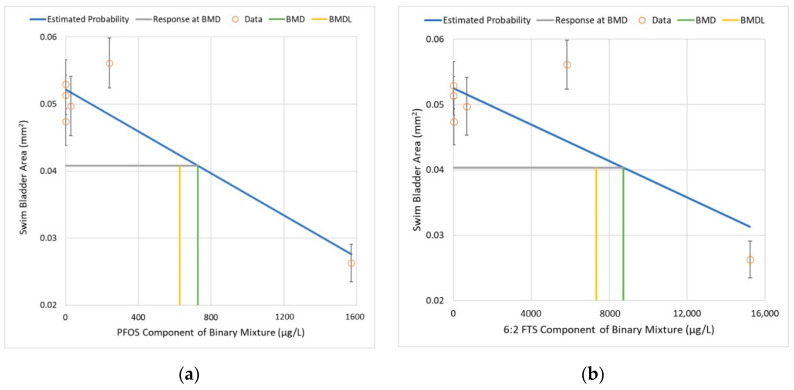
Dose–response curves for the binary mixtures experiment, showing each component chemical with SBA (all three replicates combined): (**a**) Linear D–R model fit for PFOS. (**b**) Linear D–R model fit for 6:2 FTS. See Table 6 for model parameters and relative contributions of each chemical to the total dose across a range of response levels.

The SBA results for the binary mixtures can be illustrated with similar D–R curves by plotting each component of the mixture separately (see Figure 6).

The results from the individual experiments and the binary mixtures experiment can be combined to determine the relative potency scaling factor that can be applied to 6:2 FTS to generate the equivalent PFOS dose. The following processing steps are applied:

1. Across a full % response range, calculate the corresponding PFOS dose from the individual component study (see Table 5).
2. Fit a D–R model to the PFOS component of the binary mixtures experiment and use the D–R model to predict the PFOS dose at each % response level (Figure 6a).
3. Subtract the dose from #2 (mixtures experiment) from the dose from #1 (individual component experiment); the PFOS balance remaining is presumably attributable to additional toxicity from 6:2 FTS.
4. Fit a D–R model to the 6:2 FTS component of the binary mixtures experiment and use the D–R model to predict the 6:2 FTS dose at each % response level (Figure 6b).
5. Calculate the relative potency as the ratio of the PFOS balance remaining (#3) by the predicted 6:2 FTS (#4).

**Figure 7:**
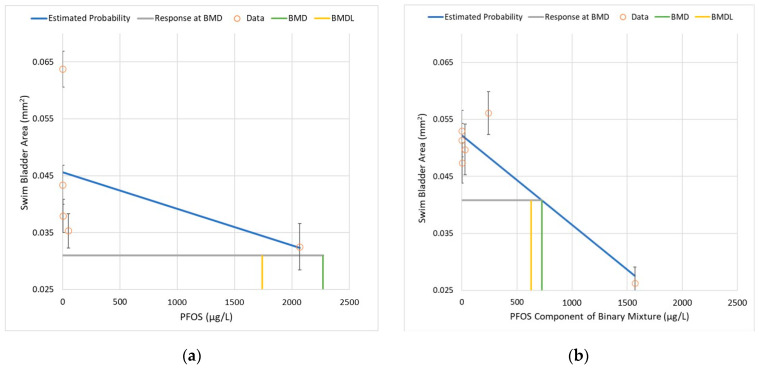
Linear D–R models fitted to PFOS datasets for (**a**) the component study and (**b**) the binary mixture study, showing differences in the slopes and intercepts. The linear D–R model parameters are (**a**) component study: intercept = 0.0456 mm^2^, slope = −6.45 × 10^−6^ mm^2^ per μg/L, and control group mean = 0.064 mm^2^; and (**b**) binary mixtures study: intercept = 0.0522 mm^2^, slope = −1.57 × 10^−5^ mm^2^ per μg/L, and control group mean = 0.053 mm^2^.

It is helpful to compare the PFOS D–R curves for the component study and the binary mixtures study side-by-side in order to further evaluate the findings in the low-dose region (e.g., <50% response) given in Table 6. Figure 7 shows the PFOS D–R curves using identical scales for the x-axis and y-axis.

With the exception of the control group and the highest dose group, the mean SBA was actually higher for the binary mixtures study for the exposure concentrations evaluated in this study. The fact that there was a lower reduction in mean SBA at concentrations of PFOS < 50 µg/L suggests that the addition of 6:2 FTS has a less-than-additive (e.g., antagonistic) interaction in the binary mixture. However, at the highest doses, in the 1000 to 2000 µg/L range for PFOS, the reduction in mean SBA was notably greater in the binary mixtures study. This finding suggests that the interaction between PFOS and 6:2 FTS is complex, cannot be generalized across all concentration ranges, and appears to transition from a less-than-additive relationship at PFOS equivalent concentrations < 100 µg/L (assuming that the addition of 6:2 FTS approximately doubles the PFOS equivalent dose for this study), to a more-than-additive relationship at higher concentrations. Without additional intermediate treatment groups, this study cannot resolve the inflection point where the transition changes from less-than-additive to more-than-additive.

**Figure 8:**
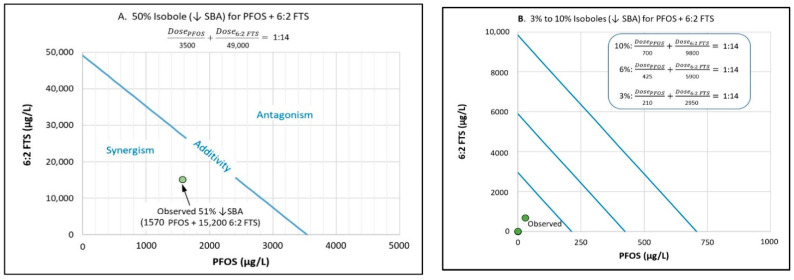
Isoboles for binary mixtures of PFOS and 6:2 FTS: Each line shows the predicted concentrations of PFOS + 6:2 FTS that, together, yield a specified response level, based on the linear dose–response models fitted to the component study results: (**A**) 50% reduction in swim bladder area (↓SBA); (**B**) approximately 3%, 6%, and 10% ↓SBA. The slope of the isobole line conveys the average relative potency (RP) across the specified dose ranges; for this study, the average RP of PFOS was approximately 14 times greater than 6:2 FTS. The assumption of dose additivity is supported if observations from the whole mixture study plot near the line; deviations convey potential interactions—synergism for points below the line (which is indicated for this study), and antagonism for points above the line [3]. Observed points are from the dose regimens for the binary mixtures experiment summarized in Table 1.

Isoboles are a useful visual aid for comparing findings from the binary mixtures experiment to the predicted response if the assumption of additivity from the component experiments holds across the dose ranges of both chemicals [3]. One limitation of isoboles is that the relative potency is assumed to be a fixed constant at each response level. Figure 8 provides examples of isoboles for selected response levels observed in the binary mixtures study. The slope of the isobole line indicates the average relative potency of the chemical plotted on the y-axis (6:2 FTS) compared to that on the x-axis (PFOS). Based on the D–R models’ fit to the component study results, the slope is 1:14 (PFOS: 6:2 FTS), which means that the potency of 6:2 FTS is estimated to be 14 times lower than that of PFOS at equivalent concentrations.

Isoboles would predict that a more-than-additive response occurs (potentially indicating synergism) for the effect on SBA at all response levels within the range 3–50%. For example, in the binary experiment, a mixture of PFOS (1570 μg/L) and 6:2 FTS (15,300 μg/L) yielded a 51% reduction in average SBA. The results of the component study for PFOS would have predicted an approximate 22% response for PFOS at 1570 μg/L as well as 6:2 FTS at 15,300 μg/L, for a sum of 44% response. The 51% response from the mixture was marginally greater (i.e., absolute difference of 7% change in mean SBA, and relative difference of 16%) than would have been predicted by the individual component studies.

### 3.5. Response Additivity

**Figure 9:**
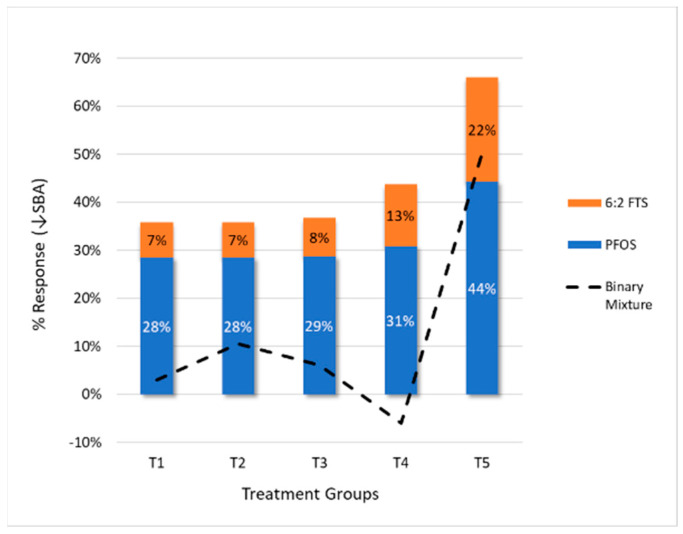
Evaluation of the assumption of response additivity for the binary mixtures study, using the linear D–R models developed from the individual experiments. The columns represent the predicted responses of both chemicals given their measured concentrations in the binary mixtures study, applied to the D–R models developed from the individual experiments. The dotted line is the actual observed response for the binary mixtures study.

Response additivity was explored by comparing the sum of the predicted responses from PFOS and 6:2 FTS measured in the binary mixtures study. The predictions were based on the linear D–R models’ fit in the individual component studies (see Section 3.2). Figure 9 shows the stacked column plot of predicted responses based on PFOS and 6:2 FTS concentrations measured in the mixtures experiment, along with the D–R functions’ fit for the individual component experiments. Overlaid on this plot is the observed response (i.e., reduction in mean SBA) in the binary mixtures study.

The results show that response addition would generally not be supported except potentially for the highest treatment group; all other responses would be overestimated by use of the D–R models fitted to data from the individual experiments. As shown in Figure 2, the mean SBA remained relatively stable, with effects < 10% until the highest dose group.

## 4. Conclusions

This study demonstrates that the relative potency of PFOS and 6:2 FTS is not constant, but instead varies as a function of dose. Under these conditions, conventional methods of calculating the ratio of PODs to estimate relative potency—such as the NOAEL/LOAEL approach—will likely fail to reproduce the observed developmental effects for mixtures of PFOS and 6:2 FTS, in some cases by many orders of magnitude. Use of the full dose–response curves from the component and whole-mixture studies is a relatively tractable and more reliable method of assessing relative potency.

In the mixtures experiment, the relative potency of PFOS to 6:2 FTS ranged from approximately 1:10 to 1:40 for changes in mean SBA of 90% to 50%, respectively (see Table 6). This is a slightly broader range than the relative potency observed in the single chemical studies, which ranged from 1:14 to 1:20 for the same response range (see Table 5). By contrast, the NOAEL/LOAEL method yielded a point estimate for relative potency of 1:20,000, while the ratio of BMDLs yielded a relative potency of approximately 1:10.

In practice, a key step in the risk assessment of mixtures is to extend findings from available toxicity study data to develop reliable estimates of dose–response for a wide range of mixture profiles where the relative magnitudes and concentrations are expected to vary from site to site. One common tactic with relative potency approaches, assuming dose additivity, is to scale the concentrations to an index chemical. This approach is applied to mixtures of PAHs and dioxins, for example, where the index chemicals are benzo(a)pyrene and 2,3,7,8 TCDD, respectively. In these approaches, the toxicity value for the index chemical forms the basis for the risk assessment of the mixture. If a similar approach were to be applied to mixtures of PFAS—using PFOS as an index chemical, for example—the use of PODs for PFOS from component studies would likely not be predictive, given the potential for interactions (both directions) observed in this study. A more reliable basis for deriving a POD for the index chemical and corresponding relative potencies of other components of the mixtures would be to use the full dose–response relationships of each component from a whole-mixture toxicity study.

This study uses the dose–response relationship for SBA as an illustrative example. Reduced swim bladder inflation may be indicative of impairment that can affect feeding and swimming behavior, as well as reproduction in later life stages [13]. The linear D–R model was selected for both component experiments and the mixture experiments in order to simplify the discussion of the methods used to derive the relative potency factor in the binary mixture experiments. However, this approach can accommodate any of the candidate dose–response models used for dichotomous and continuous variable endpoints.

Future studies with PFOS and 6:2 FTS can build upon this study by focusing on the dose range between 100 and 2000 μg/L (PFOS equivalents) to determine whether there is a predictable threshold effect within this range where the interaction shifts from less-than-additive to more-than-additive, and whether that threshold changes with ratios different from the approximate range 1:10–1:30 (PFOS to 6:2 FTS) examined in this study. In addition, concurrent evaluations of gene expression [33,34] would further elucidate potential modes of action that may explain the interactions observed with mixtures of PFOS and 6:2 FTS.

## Figures and Tables

**Table 1:** Dose regimens for component experiments and binary mixture experiments.

Treatment Group	Single Chemical Experiments ^1^		Binary Mixture ^1^	
	PFOS (μg/L) ^2^	6:2 FTS (μg/L) ^3^	PFOS (μg/L) ^4^	6:2 FTS (μg/L) ^5^
Control	0	1.9 (0)	0	0 (0)
T1	0.76 (0.1)	1.2 (1)	0.075 (0.1)	1.3 (1)
T2	3.2 (2)	23 (20)	0.63 (2)	21 (20)
T3	50 (60)	731 (600)	29 (60)	683 (600)
T4	2066 (600)	6331 (6000)	241 (600)	5825 (6000)
T5	7475 (1980)	15,530 (19,800)	1570 (1980)	15,229 (19,800)

^1^ Measured concentration (target concentration): single measurements of stock solutions were conducted; 1 microgram per liter (μg/L) equals 1 part per billion (ppb). ^2^ Molecular weight (MW) of PFOS was 500.13 μg/μM; corresponding measured concentrations (T1 to T5) were 0.00152, 0.00638, 0.0992, 4.13, and 15.0 μM. ^3^ MW of 6:2 FTS was 428.17 μg/μM; corresponding measured concentrations (T1 to T5) were 0.00285, 0.0544, 1.71, 14.8, and 36.3 μM. ^4^ Corresponding measured concentrations of PFOS: 0.00015, 0.00126, 0.058, 0.482, and 3.14 μM (see Supplementary Table S1). ^5^ Corresponding measured concentrations of 6:2 FTS: 0.00304, 0.049, 1.60, 13.6, and 35.6 μM (see Supplementary Table S1).

**Table 2:** ANOVA results (shaded squares are statistically significant, *p* < 0.05).

Group Comparison ^1^	PFOS ^2^					Binary Mixture ^2^				
	MR	HR	BL	SBA	YSA	MR	HR	BL	SBA	YSA
T0 vs. T1				L *	L *					
T0 vs. T2										
T0 vs. T3			N							
T0 vs. T4			L					N	N	
T0 vs. T5	N *	N *				N *	N *	L	L	N *

^1^ T0 = control group. T1 to T5 = treatment groups. N = NOAEL. N * = unbounded NOAEL. L = LOAEL. L * = unbounded LOAEL. ^2^ MR = mortality rate; HR = hatch rate; BL = body length; SBA = swim bladder area; YSA = yolk sac area.

**Table 3:** NOAELs, LOAELs, and POD ratios for PFOS and 6:2 FTS.

POD	Effect Endpoint	PFOS (μg/L) ^1^	6:2 FTS (μg/L) ^2^	PFOS/ 6:2 FTS ^3^	6:2 FTS/ PFOS ^4^
NOAEL	BL	50	>15,530	<3.2 × 10^−3^	>310
	SBA	<0.76	>15,530	<4.9 × 10^−5^	>20,400
	YSA	<0.76	>15,530	<4.9 × 10^−5^	>20,400
LOAEL	BL	2066	>15,530	<1.3 × 10^−1^	>7.5
	SBA	<0.76	>15,530	<4.9 × 10^−5^	>20,400
	YSA	<0.76	>15,530	<4.9 × 10^−5^	>20,400

^1^ For PFOS, the difference in mean SBA and YSA is statistically significant for all treatment groups, so T1 (0.76 μg/L) defines the unbounded LOAEL; although this study does not provide a direct estimate of an NOAEL, presumably, NOAEL < LOAEL < 0.76 μg/L. ^2^ For 6:2 FTS, none of the treatment groups are statistically significant for any of the effect endpoints, so T5 (15,530 μg/L) defines the unbounded NOAEL; although this study does not provide a direct estimate of an LOAEL, presumably, 15,530 μg/L < NOAEL < LOAEL. ^3^ The relative potency of 6:2 FTS compared to PFOS is given by the ratio of PODs expressed as PFOS/6:2 FTS. All results are “<” because the numerator is “<” for all but BL, and the denominator is “>”. ^4^ PFOS potency relative to 6:2 FTS, rounded to two significant digits. All results are “>” because the numerator is “>” and the denominator is “<” for all but BL.

**Table 4:** BMD and BMDL estimates for PFOS for the SBA endpoint.

Dose–Response Model	BMD (μg/L)	BMDL (μg/L)	AIC	Scaled Residual at Dose Group Near BMR
Linear	2268	1739	−1155.8	0.097
Power ^1^	2268	1732	−1155.8	0.097
Polynomial ^1^	2268	1744	−1155.8	0.097
Exponential ^2^	2287	1642	−1156.0	0.136
Hill	0.2 ^3^	0.1 ^3^	−1277.5	0.023

^1^ Power and second-order polynomial models overlap with the linear model, and are excluded from Figure 3. ^2^ Example is for a 3-parameter exponential model; the 2-parameter exponential model overlaps with the linear model. ^3^ BMDS indicates that the result for the Hill model is questionable because the BMD is more than three times lower than the lowest dose (0.76 µg/L).

**Table 5:** Predicted doses of PFOS and 6:2 FTS (applied individually, rather than as a mixture) that yield the same response (reduction in mean SBA).

% Response	PFOS (μg/L) ^1^	6:2 FTS (μg/L) ^2^	PFOS/ 6:2 FTS	6:2 FTS/ PFOS
20%	-- ^3^	13,362	-- ^3^	-- ^3^
30%	158	23,973	0.007	151
40%	1147	34,583	0.033	30.1
50%	2136	45,193	0.047	21.2
60%	3125	55,804	0.056	17.9
70%	4113	66,414	0.062	16.1
80%	5102	77,025	0.066	15.1
90%	6091	87,635	0.070	14.4

^1^ For PFOS, the parameters of the linear D–R model are a = 0.04563 mm^2^, b = −6.45 × 10^−6^ mm^2^ per μg/L, and control group mean = 0.0637 mm^2^, corresponding to a 16% response at the intercept. ^2^ For 6:2 FTS, the parameters of the linear D–R model are a = 0.05499 mm^2^, b = −5.60 × 10^−7^ mm^2^ per μg/L, and control group mean = 0.0594 mm^2^, corresponding to an 18% response at the intercept. ^3^ The intercept term of the linear D–R model (i.e., predicted response at PFOS = 0) begins at approximately 27% lower mean SBA than the observed control group mean.

**Table 6:** Estimate of the PFOS equivalent dose to determine the relative potency factor for 6:2 FTS from the binary mixture experiment.

% Response	PFOS Only (μg/L)	PFOS in Mixture (μg/L) ^1^	PFOS Balance (μg/L)	6:2 FTS in Mixture (μg/L) ^2^	PFOS/ 6:2 FTS	6:2 FTS/ PFOS
20%	-- ^3^	627	-- ^3^	7287	-- ^3^	-- ^3^
30%	158	965	-- ^4^	11,091	-- ^4^	-- ^4^
40%	1147	1303	-- ^4^	14,895	-- ^4^	-- ^4^
50%	2136	1641	495	18,699	0.026	37.8
60%	3125	1979	1146	22,503	0.051	19.6
70%	4113	2317	1796	26,307	0.068	14.6
80%	5102	2656	2446	30,111	0.081	12.3
90%	6091	2994	3097	33,915	0.091	11.0

^1^ For PFOS in the binary mixtures, the parameters of the linear D–R model are a = 0.05218 mm^2^, b = −1.57 × 10^−5^ mm^2^ per μg/L, and control group mean = 0.0529 mm^2^, corresponding to a 21% response at the intercept. ^2^ For 6:2 FTS, the parameters of the linear D–R model are a = 0.05250 mm^2^, b = −1.39 × 10^−6^ mm^2^ per μg/L, and control group mean = 0.0529 mm^2^, corresponding to a 21% response at the intercept. ^3^ The intercept term of the linear D–R model (i.e., predicted response at PFOS = 0) begins at approximately 27% lower mean SBA than the observed control group mean. ^4^ The PFOS concentration in the binary mixtures is greater than the PFOS concentration of the component study at the same response level, suggesting that the interaction with 6:2 FTS is antagonistic.

## Data Availability

Data are provided in the Supplementary Materials.

## Supplementary Materials

The following supporting information can be downloaded at: https://www.mdpi.com/article/10.3390/toxics10080418/s1, Table S1: Nominal and measured concentrations of PFOS and 6:2 FTS in stock solutions; Table S2: Mortality rate and hatch rate by experiment, treatment, replicate, and days post-fertilization; Table S3: Morphological measurements by experiment, treatment, and replicate; Table S4: Results of pairwise comparisons for effects on mean SBA using the R package emmeans; Table S5: Relative potency of PFOS and 6:2 FTS to aquatic organisms based on matched test species, effect endpoints, and points of departure; Table S6: Rank order of points of departure for zebrafish toxicity studies with PFOS, including results from this study; Figure S1: Cumulative probability distribution of *n* = 24 points of departure from zebrafish toxicity studies with PFOS. (References [35,36,37,38,39,40] are cited in the Supplementary Materials).
