# Supplementary material for: Is Mixtures’ Additivity Supported by Empirical Data? A Case Study of Developmental Toxicity of PFOS and 6:2 FTS in Wildtype Zebrafish Embryos
Source: Toxics. 2022 Jul 25;10(8):418. doi: 10.3390/toxics10080418 (PMC9329885; doi:10.3390/toxics10080418)
Supplement: Supplementary file 1 [file toxics-10-00418-s001.zip › toxics-1741770-SM.pdf]

toxics

MDPI

# Supplementary Material: Is Mixtures Additivity Supported by Empirical Data? A Case Study of Developmental Toxicity of PFOS and 6:2FTS in Wildtype Zebrafish Embryos

**Megan E. Fey, Philip E. Goodrum, N. Roxanna Razavi, Christopher M. Whipps, Sujan Fernando and Janet K. Anderson [2]**

**Table S1.** Relative potency of PFOS and 6:2 FTS to aquatic organisms based on matched test species, effect endpoints, and points of departure (POD).

| Test Species | Study Duration (days) | Effect Endpoint | POD | Concentration (µg/L)[1] | | Relative Potency[2] | Ref. |
|---|---|---|---|---|---|---|---|
| | | | | PFOS | 6:2 FTS | | |
| Eastern Tiger Salamander *Ambystoma tigrinum* | 31 | body condition | LOAEL[3] | >1000 | <10 | <1:0.01 | [35,36] |
| Eastern Tiger Salamander *Ambystoma tigrinum* | 31 | snout vent length | LOAEL | 100 | <10 | <1:0.1 | [35,36] |
| Water Flea *Daphnia magna* | 2 | immobility/ lethality | $EC_{50}$ | 672,000 | >109,000 | >1:0.2 | [37,38] |
| Northern Leopard Frog *Lithobates pipiens* | 40 | developmental delay | LOAEL | 200 | >1000 | >1:5 | [35,36] |
| Rainbow Trout *Oncorhynchus mykiss* | 4 | survival | $LC_{50}$ | 22,000 | >107,000 | >1:5 | [37,38,39] |
| Zebrafish, *Danio rerio* | 7 | survival | $LC_{50}$ | 1000 | 8000 | 1:8 | [40] |
| Moor Frog, *Rana arvalis* | 7 | survival | $LC_{50}$ | 1400 | 10,500 | 1:8 | [40] |
| American Toad *Anaxyrus americanus* | 30 | snout vent length & body mass | NOAEL | 100 | >1000 | >1:10 | [35,36] |
| Northern Leopard Frog *Lithobates pipiens* | 40 | developmental delay | NOAEL | 10 | 1000 | 1:100 | [35,36] |

$EC_{50}$ = effective concentration that gives half-maximal response; LOAEL= lowest observed adverse effect level; POD = point of departure; $LC_{50}$ = lethal concentration that causes 50% mortality; NOAEL= no observed adverse effect level; [1] Values with ">" are unbounded for the study. Unbounded NOAELs are the highest treatment group tested. Unbounded LOAELs are the highest treatment group tested; [2] Relative potency based on the ratio of PODs (i.e., 6:2 FTS / PFOS). Results with ">" reflect an unbounded NOAEL or LC50 for 6:2 FTS (numerator) or unbounded LOAEL for PFOS (denominator). Results with "<" reflect an unbounded NOAEL for PFOS (denominator) or unbounded LOAEL for 6:2 FTS (numerator); [3] PFOS toxicity study for *A. tigrinum* supports an unbounded NOAEL for PFOS, meaning the NOAEL is > 1,000 µg/L, and the LOAEL would occur at an even higher concentration.

**Table S2.** Nominal and measured concentrations of PFOS and 6:2 FTS in stock solutions used for three experiments: 1) PFOS; 2) 6:2 FTS; and 3) binary mixtures of PFOS + 6:2 FTS at a target 1:10 concentration ratio. Values are presented for each experiment/chemical group in units of µg/L (top) and µM (bottom). Molecular weights (g/mol or µg/µM) are 500.13 for PFOS (CASRN 1763-23-1) and 428.17 for 6:2 FTS (CASRN 27619-97-2).

| **Experiment & Chemical** | | **Control** | | **T1** | | **T2** | | **T3** | | **T4** | | **T5** | |
|---|---|---|---|---|---|---|---|---|---|---|---|---|---|
| | | **N** | **M** | **N** | **M** | **N** | **M** | **N** | **M** | **N** | **M** | **N** | **M** |
| **Concentration (µg/L)** | | | | | | | | | | | | | |
| 1- PFOS | PFOS | 0 | 0 | 0.10 | 0.76 | 2.0 | 3.2 | 60 | 49.6 | 600 | 2070 | 1980 | 7480 |
| | 6:2 FTS | 0 | 0 | 0 | 0 | 0 | 0 | 0 | 0 | 0 | 0 | 0 | 0 |
| 2- 6:2 FTS | PFOS | 0 | 0 | 0 | 0.028 | 0 | 0 | 0 | 0 | 0 | 0 | 0 | 0 |
| | 6:2 FTS | 0 | 1.9 | 1.0 | 1.2 | 20.0 | 23.3 | 600 | 731 | 6000 | 6,330 | 19,800 | 15,500 |
| 3- PFOS + 6:2 FTS | PFOS | 0 | 0 | 0.10 | 0.075 | 2.0 | 0.63 | 60 | 29.0 | 600 | 241 | 1980 | 1570 |
| | 6:2 FTS | 0 | 0 | 1.0 | 1.3 | 20.0 | 21.0 | 600 | 683 | 6000 | 5,830 | 19,800 | 15,300 |
| | Ratio[1] | 0 | 0 | 1:10 | 1:17 | 1:10 | 1:33 | 1:10 | 1:24 | 1:10 | 1:24 | 1:10 | 1:10 |
| **Concentration (µM)** | | | | | | | | | | | | | |
| 1- PFOS | PFOS | 0 | 0 | 0.0002 | 0.0015 | 0.0040 | 0.0064 | 0.120 | 0.099 | 1.2 | 4.1 | 4.0 | 15.0 |
| | 6:2 FTS | 0 | 0 | 0 | 0 | 0 | 0 | 0 | 0 | 0 | 0 | 0 | 0 |
| 2- 6:2 FTS | PFOS | 0 | 0 | 0 | 0.00006 | 0 | 0 | 0 | 0 | 0 | 0 | 0 | 0 |
| | 6:2 FTS | 0 | 0.0045 | 0.0023 | 0.00285 | 0.0467 | 0.544 | 1.4 | 1.7 | 14.0 | 14.8 | 46.2 | 36.3 |
| 3- PFOS + 6:2 FTS | PFOS | 0 | 0 | 0.0002 | 0.00015 | 0.004 | 0.00126 | 0.12 | 0.058 | 1.2 | 0.48 | 4.0 | 3.1 |
| | 6:2 FTS | 0 | 0 | 0.0023 | 0.00304 | 0.0467 | 0.049 | 1.4 | 1.6 | 14.0 | 13.6 | 46.2 | 35.6 |

N = nominal concentration; M = measured concentration.

1. Ratio = 6:2 FTS / PFOS, equivalently expressed as the proportion PFOS: 6:2 FTS. The nominal concentration ratio for each treatment group of the binary experiment was 1 part PFOS per 10 parts 6:2 FTS (1:10). Measured concentration ratios ranged from 1:10 to 1:33.

**Table S3.** Mortality rate and hatch rate by experiment, treatment, replicate, and days post fertilization.

| | | | Count[1] | | 1 dpf | | 2 dpf | | 3 dpf | | 4 dpf | | 5 dpf | |
|---|---|---|---|---|---|---|---|---|---|---|---|---|---|---|
| **Experiment** | **Treatment** | **Replicate** | **Start** | **End** | **MR** | **HR** | **MR** | **HR** | **MR** | **HR** | **MR** | **HR** | **MR** | **HR** |
| 1- PFOS | 0 | A | 20 | 15 | 25% | 0% | 25% | 0% | 25% | 75% | 25% | 75% | 25% | 75% |
| | 0 | B | 20 | 13 | 35% | 0% | 35% | 0% | 35% | 65% | 35% | 65% | 35% | 65% |
| | 0 | C | 20 | 15 | 30% | 0% | 30% | 5% | 25% | 75% | 25% | 75% | 25% | 75% |
| | 1 | A | 20 | 14 | 30% | 0% | 30% | 0% | 30% | 70% | 30% | 70% | 30% | 70% |
| | 1 | B | 20 | 15 | 25% | 0% | 25% | 0% | 25% | 75% | 25% | 75% | 25% | 75% |
| | 1 | C | 20 | 13 | 35% | 0% | 35% | 0% | 35% | 65% | 35% | 65% | 35% | 65% |
| | 2 | A | 20 | 15 | 25% | 0% | 25% | 0% | 25% | 75% | 25% | 75% | 25% | 75% |
| | 2 | B | 20 | 12 | 35% | 0% | 35% | 0% | 40% | 60% | 40% | 60% | 40% | 60% |
| | 2 | C | 19 | 11 | 32% | 0% | 32% | 5% | 42% | 63% | 42% | 63% | 42% | 63% |
| | 3 | A | 20 | 15 | 35% | 0% | 35% | 5% | 25% | 75% | 25% | 75% | 25% | 75% |
| | 3 | B | 20 | 13 | 35% | 0% | 35% | 0% | 35% | 65% | 35% | 65% | 35% | 65% |
| | 3 | C | 20 | 11 | 45% | 0% | 45% | 0% | 45% | 55% | 45% | 55% | 45% | 55% |
| | 4 | A | 20 | 13 | 35% | 0% | 35% | 0% | 35% | 65% | 35% | 65% | 35% | 65% |
| | 4 | B | 20 | 18 | 10% | 0% | 10% | 0% | 10% | 90% | 10% | 90% | 10% | 90% |
| | 4 | C | 20 | 17 | 15% | 0% | 15% | 25% | 15% | 85% | 15% | 85% | 15% | 85% |
| | 5 | A | 20 | 17 | 15% | 0% | 15% | 0% | 15% | 85% | 15% | 85% | 15% | 85% |
| | 5 | B | 20 | 12 | 40% | 0% | 40% | 0% | 40% | 60% | 40% | 60% | 40% | 60% |
| | 5 | C | 20 | 13 | 35% | 0% | 35% | 0% | 35% | 65% | 35% | 65% | 35% | 65% |
| 2- 6:2 FTS | 0 | A | 20 | 7 | 60% | 0% | 60% | 0% | 65% | 40% | 65% | 40% | 65% | 40% |
| | 0 | B | 20 | 8 | 55% | 0% | 55% | 0% | 60% | 40% | 60% | 40% | 60% | 40% |
| | 0 | C | 20 | 9 | 50% | 0% | 55% | 0% | 55% | 45% | 55% | 45% | 55% | 45% |
| | 1 | A | 20 | 7 | 60% | 0% | 65% | 5% | 65% | 35% | 65% | 35% | 65% | 35% |
| | 1 | B | 20 | 4 | 55% | 0% | 80% | 5% | 80% | 20% | 80% | 20% | 80% | 20% |
| | 1 | C | 20 | 13 | 50% | 0% | 35% | 25% | 35% | 65% | 35% | 65% | 35% | 65% |
| | 2 | A | 21 | 6 | 62% | 0% | 71% | 0% | 71% | 29% | 71% | 29% | 71% | 29% |
| | 2 | B | 20 | 6 | 60% | 0% | 65% | 5% | 70% | 30% | 70% | 30% | 70% | 30% |
| | 2 | C | 20 | 9 | 50% | 0% | 55% | 40% | 55% | 45% | 55% | 45% | 55% | 45% |
| | 3 | A | 20 | 6 | 60% | 0% | 70% | 0% | 70% | 30% | 70% | 30% | 70% | 30% |
| | 3 | B | 20 | 7 | 65% | 0% | 65% | 5% | 65% | 35% | 65% | 35% | 65% | 35% |
| | 3 | C | 20 | 5 | 75% | 0% | 75% | 5% | 75% | 25% | 75% | 25% | 75% | 25% |
| | 4 | A | 20 | 6 | 65% | 0% | 70% | 5% | 70% | 30% | 70% | 30% | 70% | 30% |
| | 4 | B | 20 | 7 | 60% | 0% | 65% | 0% | 65% | 35% | 65% | 35% | 65% | 35% |
| | 4 | C | 20 | 8 | 55% | 0% | 60% | 5% | 60% | 40% | 60% | 40% | 60% | 40% |
| | 5 | A | 20 | 7 | 65% | 0% | 65% | 0% | 65% | 35% | 65% | 35% | 65% | 35% |
| | 5 | B | 20 | 5 | 70% | 0% | 75% | 0% | 75% | 25% | 75% | 25% | 75% | 25% |
| | 5 | C | 20 | 6 | 65% | 0% | 65% | 0% | 70% | 30% | 70% | 30% | 70% | 30% |
| 3- PFOS + 6:2 FTS | 0 | A | 20 | 10 | 50% | 0% | 50% | 5% | 50% | 50% | 50% | 50% | 50% | 50% |
| | 0 | B | 20 | 14 | 20% | 0% | 20% | 10% | 25% | 75% | 30% | 75% | 25% | 75% |
| | 0 | C | 20 | 15 | 25% | 0% | 25% | 5% | 25% | 75% | 25% | 75% | 25% | 75% |
| | 1 | A | 20 | 13 | 30% | 0% | 30% | 5% | 30% | 70% | 35% | 70% | 35% | 70% |
| | 1 | B | 20 | 14 | 25% | 0% | 25% | 5% | 30% | 70% | 30% | 70% | 30% | 70% |
| | 1 | C | 20 | 10 | 40% | 0% | 40% | 50% | 40% | 60% | 50% | 60% | 50% | 60% |
| | 2 | A | 20 | 12 | 35% | 0% | 40% | 5% | 40% | 60% | 40% | 60% | 40% | 60% |
| | 2 | B | 20 | 16 | 15% | 0% | 15% | 20% | 15% | 85% | 20% | 85% | 20% | 85% |
| | 2 | C | 20 | 14 | 30% | 0% | 30% | 35% | 30% | 70% | 30% | 70% | 30% | 70% |
| | 3 | A | 20 | 10 | 35% | 0% | 40% | 0% | 50% | 60% | 50% | 60% | 50% | 60% |
| | 3 | B | 21 | 10 | 52% | 0% | 52% | 14% | 52% | 48% | 52% | 48% | 52% | 48% |

| Experiment | Treatment | Replicate | Count[1] Start | End | 1 dpf MR | HR | 2 dpf MR | HR | 3 dpf MR | HR | 4 dpf MR | HR | 5 dpf MR | HR |
|---|---|---|---|---|---|---|---|---|---|---|---|---|---|---|
| | 3 | C | 21 | 13 | 38% | 0% | 38% | 10% | 38% | 62% | 38% | 62% | 38% | 62% |
| | 4 | A | 19 | 12 | 37% | 0% | 37% | 5% | 37% | 63% | 37% | 63% | 37% | 63% |
| | 4 | B | 20 | 15 | 25% | 0% | 25% | 0% | 25% | 75% | 25% | 75% | 25% | 75% |
| | 4 | C | 20 | 15 | 25% | 0% | 25% | 5% | 25% | 75% | 25% | 75% | 25% | 75% |
| | 5 | A | 19 | 12 | 37% | 0% | 37% | 11% | 37% | 63% | 37% | 63% | 37% | 63% |
| | 5 | B | 20 | 13 | 25% | 0% | 30% | 10% | 30% | 70% | 35% | 70% | 30% | 70% |
| | 5 | C | 20 | 11 | 45% | 0% | 45% | 15% | 45% | 55% | 45% | 55% | 45% | 55% |

dpf = days post fertilization; HR = hatch rate; MR = mortality rate.

1. Counts began after the eggs were dispensed into the Petri dishes.

**Table S4.** Morphological measurements by experiment, treatment, and replicate.

| Experiment | Treatment | Replicate | Body Length (mm) | Swim Bladder Area ($mm^2$) | Yolk Sac Area ($mm^2$) |
|---|---|---|---|---|---|
| 1- PFOS | 0 | A | 3.99 | 0.0575 | 0.087 |
| | 0 | B | 3.83 | 0.0704 | 0.0943 |
| | 0 | C | 3.82 | 0.0638 | 0.0894 |
| | 1 | A | 3.75 | 0.0497 | 0.0385 |
| | 1 | B | 3.81 | 0.0385 | 0.0341 |
| | 1 | C | 3.85 | 0.0421 | 0.0459 |
| | 2 | A | 3.84 | 0.0348 | 0.0469 |
| | 2 | B | 3.84 | 0.041 | 0.0402 |
| | 2 | C | 4.04 | 0.0388 | 0.0425 |
| | 3 | A | 3.7 | 0.033 | 0.0597 |
| | 3 | B | 3.69 | 0.0356 | 0.0558 |
| | 3 | C | 3.72 | 0.0382 | 0.0496 |
| | 4 | A | 3.63 | 0.0371 | 0.0669 |
| | 4 | B | 3.53 | 0.0333 | 0.0628 |
| | 4 | C | 3.58 | 0.0281 | 0.0583 |
| | 5 | A | 3.75 | 0.0485 | 0.0869 |
| | 5 | B | 3.71 | 0.0506 | 0.0813 |
| | 5 | C | 3.65 | 0.0424 | 0.0742 |
| 2- 6:2 FTS | 0 | A | 3.9 | 0.0617 | 0.0601 |
| | 0 | B | 3.73 | 0.0571 | 0.0649 |
| | 0 | C | 3.69 | 0.0597 | 0.0523 |
| | 1 | A | 3.84 | 0.053 | 0.0562 |
| | 1 | B | 3.7 | 0.0583 | 0.0615 |
| | 1 | C | 3.65 | 0.0516 | 0.0529 |
| | 2 | A | 3.73 | 0.0473 | 0.0629 |
| | 2 | B | 3.68 | 0.0568 | 0.0627 |
| | 2 | C | 3.8 | 0.0544 | 0.0737 |
| | 3 | A | 3.62 | 0.0572 | 0.0565 |
| | 3 | B | 3.81 | 0.0549 | 0.0587 |
| | 3 | C | 3.59 | 0.0483 | 0.0652 |
| | 4 | A | 3.61 | 0.0531 | 0.0612 |
| | 4 | B | 3.99 | 0.0434 | 0.0577 |
| | 4 | C | 3.83 | 0.0558 | 0.0639 |
| | 5 | A | 3.59 | 0.0427 | 0.0569 |
| | 5 | B | 3.71 | 0.041 | 0.0561 |
| | 5 | C | 3.76 | 0.0559 | 0.0562 |

| Experiment | Treatment | Replicate | Body Length (mm) | Swim Bladder Area ($mm^2$) | Yolk Sac Area ($mm^2$) |
|---|---|---|---|---|---|
| 3- PFOS + 6:2 FTS | 0 | A | 3.77 | 0.0531 | 0.0655 |
| | 0 | B | 3.64 | 0.0524 | 0.0634 |
| | 0 | C | 3.64 | 0.0534 | 0.0526 |
| | 1 | A | 3.6 | 0.0493 | 0.0784 |
| | 1 | B | 3.79 | 0.0527 | 0.0507 |
| | 1 | C | 3.63 | 0.0521 | 0.0523 |
| | 2 | A | 3.77 | 0.0473 | 0.0638 |
| | 2 | B | 3.66 | 0.0491 | 0.0541 |
| | 2 | C | 3.74 | 0.0454 | 0.057 |
| | 3 | A | 3.82 | 0.055 | 0.0614 |
| | 3 | B | 3.75 | 0.0489 | 0.0616 |
| | 3 | C | 3.53 | 0.0463 | 0.0601 |
| | 4 | A | 3.67 | 0.0564 | 0.0799 |
| | 4 | B | 3.69 | 0.054 | 0.0779 |
| | 4 | C | 3.56 | 0.0703 | 0.0614 |
| | 5 | A | 3.24 | 0.0319 | 0.072 |
| | 5 | B | 3.45 | 0.026 | 0.0797 |
| | 5 | C | 3.32 | 0.0204 | 0.064 |

**Table S5.** Results of pairwise comparisons for effects on mean SBA using R package *emmeans*.

| Experiment | Treatment (μg/L)[1] | Emmean [LCL, UCL][2] sq mm | Contrast with T0 sq mm[3] | Contrast with T0 % change[4] | df | T Ratio | *p*-value[5] |
|---|---|---|---|---|---|---|---|
| 1-PFOS | T0 (0.00) | 0.0639 [0.0581, 0.0697] | N/A | N/A | N/A | N/A | N/A |
| | **T1 (0.76)** | 0.0434 [0.0376, 0.0493] | 0.02047 | 32% | 12 | 5.396 | **0.002** |
| | **T2 (3.2)** | 0.0382 [0.0324, 0.0440] | 0.02570 | 40% | 12 | 6.775 | **< 0.001** |
| | **T3 (49.6)** | 0.0356 [0.0298, 0.0414] | 0.02830 | 44% | 12 | 7.261 | **< 0.001** |
| | **T4 (2,070)** | 0.0328 [0.0270, 0.0387] | 0.03107 | 49% | 12 | 8.190 | **< 0.001** |
| | **T5 (7,480)** | 0.0472 [0.0413, 0.0530] | 0.01673 | 26% | 12 | 4.411 | **0.009** |
| 2-6:2 FTS | T0 (1.9) | 0.0595 [0.0528, 0.0662] | N/A | N/A | N/A | N/A | N/A |
| | T1 (1.2) | 0.0543 [0.0476, 0.0610] | 0.00520 | 9% | 12 | 1.187 | 0.83 |
| | T2 (23.3) | 0.0528 [0.0461, 0.0596] | 0.00667 | 11% | 12 | 1.522 | 0.66 |
| | T3 (731) | 0.0535 [0.0467, 0.0602] | 0.00603 | 10% | 12 | 1.378 | 0.74 |
| | T4 (6,330) | 0.0508 [0.0440, 0.0575] | 0.00873 | 15% | 12 | 1.994 | 0.40 |
| | T5 (15,500) | 0.0465 [0.0398, 0.0533] | 0.01297 | 22% | 12 | 2.961 | 0.10 |
| 3-PFOS + 6:2 FTS | T0 (0 + 0) | 0.0530 [0.0469, 0.0590] | N/A | N/A | 12 | N/A | N/A |
| | T1 (0.075 + 1.3) | 0.0514 [0.0453, 0.0574] | 0.00160 | 3% | 12 | 0.409 | 0.99 |
| | T2 (0.63 + 21.0) | 0.0473 [0.0412, 0.0533] | 0.00570 | 11% | 12 | 1.459 | 0.69 |
| | T3 (29.0 + 683) | 0.0501 [0.0440, 0.0561] | 0.00290 | 5% | 12 | 0.7452 | 0.97 |
| | T4 (241 + 5,830) | 0.0602 [0.0542, 0.0663] | −0.00727 | −14% | 12 | −1.860 | 0.47 |
| | **T5 (1,570 + 15,300)** | 0.0261 [0.0201, 0.0321] | 0.02687 | 51% | 12 | 6.875 | **< 0.001** |

N/A = not applicable; pairwise contrasts are T1 through T5 vs T0; SBA = swim bladder area; sq mm = square millimeters; [1] T0 = control group. Concentrations (in parentheses) of treatments increase from T1 to T5; [2] Confidence level = 0.95. Pooled standard error for the mean is 0.00276 $mm^2$; [3] Contrast with T0 is the difference in means: [T0 – Ti]. Pooled standard error for the mean of contrasts is 0.00391 $mm^2$; [4] Percent change is 100% x [T0-Ti]/T0; [5] Statistically significant contrasts ($p < 0.05$) are highlighted in bold.

**Table S6.** Rank order of points of departure (POD) for zebrafish toxicity studies with PFOS including results from this study (bold font).

| Rank | Study | Endpoint | Abbreviation | PFOS (μg/L) | POD[1] |
|---|---|---|---|---|---|
| 1.5 | **This study** | **swim bladder area** | **SBA** | **0.76** | **LOAEL*** |
| 1.5 | **This study** | **yolk sac area** | **YSA** | **0.76** | **LOAEL*** |
| 3 | **This study** | **body length** | **BL** | **50** | **NOAEL** |
| 4 | Huang et al., 2010 [41] | cell death, swimming behavior, malformations | Development | 1120 | $EC_{50}$ |
| 5 | **This study** | **swim bladder area** | **SBA** | **1739** | **BMDL** |
| 6 | Vogs et al., 2019 [42] | uninflated swim bladder and curved spine | SBA | 1900 | $EC_{50}$ |
| 7.5 | Jantzen et al., 2016 [43] | body length | BL | 2000 | LOAEL |
| 7.5 | Jantzen et al., 2016 [43] | yolk sac area | YSA | 2000 | LOAEL |
| 9 | **This study** | **body length** | **BL** | **2066** | **LOAEL** |
| 10 | Huang et al., 2010 [41] | mortality rate | MR | 2200 | $LC_{50}$ |
| 11 | Hagenaars et al., 2014 [44] | inflated swim bladder | SBA | 2290 | $EC_{50}$ |
| 12 | Martinez et al., 2019 [45] | swim bladder inflation | SBA | 2500 | LOAEL |
| 13.5 | Martinez et al., 2019 [45] | body length | BL | 5000 | LOAEL |
| 13.5 | Martinez et al., 2019 [45] | yolk sac area | YSA | 5000 | LOAEL |
| 15 | Hagenaars et al., 2014 [44] | developmental lethality | MR | 6250 | $LC_{50}$ |
| 16 | Sant et al., 2017 [46] | exocrine pancreas morphology | Development | 8000 | LOAEL |
| 17 | Kherzi et al., 2017 [47] | uninflated swim bladder, less developed gut, curved spine | SBA | 8002 | LOAEL |
| 18 | Martinez et al., 2019 [45] | mortality rate | MR | 9100 | $LC_{50}$ |
| 19.5 | Annunziato et al., 2020 [48] | body length | BL | 17,640 | LOAEL |
| 19.5 | Annunziato et al., 2020 [48] | yolk sac area | YSA | 17,640 | LOAEL |
| 21 | Annunziato et al., 2020 [48] | developmental lethality | MR | 31,000 | $LC_{50}$ |
| 22 | Ding et al., 2013 [49] | mortality | MR | 50,510 | $LC_{50}$ |
| 23.5 | Ding et al., 2013 [49] | mortality rate | MR | 54,400 | $LC_{50}$ |
| 23.5 | Ding et al., 2013 [49] | mortality rate | MR | 54,900 | $LC_{50}$ |

$EC_{50}$ = effective concentration that gives half-maximal response; LOAEL= lowest observed adverse effect level; POD = point of departure; $LC_{50}$ = lethal concentration that causes 50% mortality; NOAEL= no observed adverse effect level; [1] This study yields unbounded LOAELs (shown as LOAEL*) for SBA and YSA.

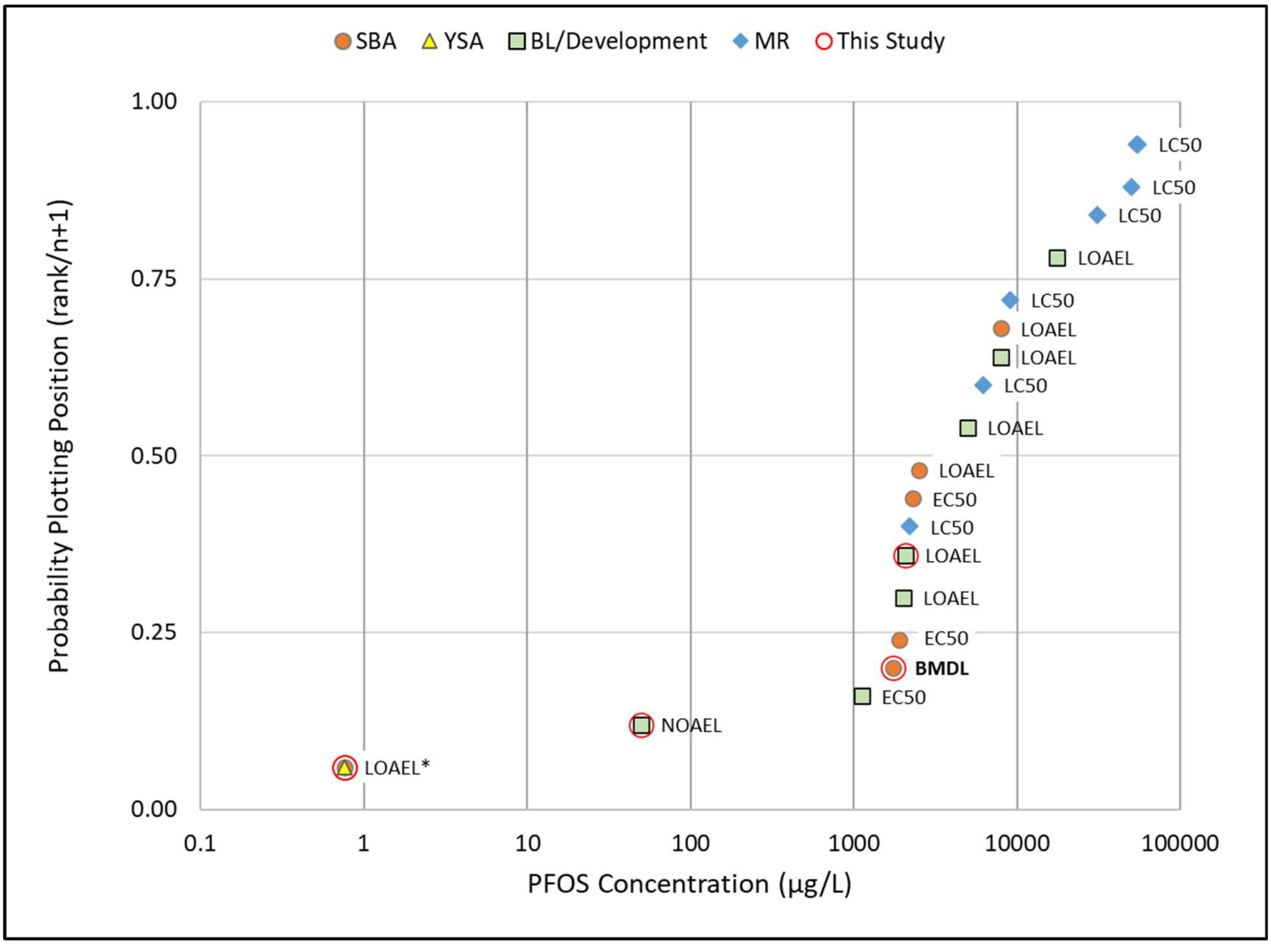


**Figure S1.** Cumulative probability distribution of n=24 points of departure (POD) from zebrafish toxicity studies with PFOS (see Table S6) shown on a log base 10 scale. SBA = swim bladder area, YSA = yolk sac area, BL = body length, MR = mortality rate. Selected PODs from this study are highlighted with a red circle. The benchmark dose lower bound (BMDL) for this study, approximately 1,700 µg/L, corresponds to the 20$^{th}$ percentile. The BMDL is the 95% lower confidence limit for the benchmark response at one control group standard deviation, and is most analagous to a NOAEL POD. LOAEL* is an unbounded LOAEL for SBA and YSA from this study.

## References


35. Hoover, G.M.; Purdue University; Forestry and Natural Resources. Effects of Per/Polyfluoroalkyl Substance Exposure on Larval Amphibians; ProQuest Dissertations & Theses: Ann Arbor, **2018**.
36. Flynn, W., Hoover, G., Iacchetta, M., Guffey, S., Li, W., Hoverman, J.T., Lee, L.S., Sepulveda, M.S. Comparative Toxicity of Aquatic Per- and Polyfluoroalkyl Substance Exposure in Three Species of Amphibians. Purdue University Research Repository. **2022**. doi:10.4231/GGPV-C321.
37. Hoke, R.A.; Ferrell, B.D.; Ryan, T.; Sloman, T.L.; Green, J.W.; Nabb, D.L.; Mingoia, R.; Buck, R.C.; Korzeniowski, S.H. Aquatic hazard, bioaccumulation and screening risk assessment for 6: 2 fluorotelomer sulfonate. *Chemosphere*, **2015**, *128*, 258–265.
38. Palmer, S.J.; Van Hoven, R.L.; Krueger, H.O. Perfluorooctanesulfonate, Potassium Salt (PFOS): A 96-hr Static Acute Toxicity Test with the Rainbow Trout (*Oncorhynchus mykiss*). Report No. 454A-145. EPA Docket AR226-1030a044. Wildlife International Ltdl, Easton, MD.
39. Beach, S.A; Newsted, J.L.; Coady, K., Giesy, J.P. Ecotoxicological evaluation of perfluorooctanesulfonate (PFOS). *Rev Environ Contam Toxicol*, **2006**, *186*, 133–174.
40. Norström, K.; Viktor, T.; Palm Cousins, A.; Rahmberg, M. Risks and Effects of the Dispersion of PFAS on Aquatic, Terrestrial and Human Populations in the Vicinity of International Airports; B 2232; Swedish Environmental Research Institute, **2015**.
41. Huang, H.; Huang, C.; Wang, L.; Ye, X.; Bai, C.; Simonich, M.T.; Tanguay, R.L.; Dong, Q. Toxicity, uptake kinetics and behavior assessment in zebrafish embryos following exposure to perfluorooctanesulphonic acid (PFOS). *Aq. Tox*. **2010**, *98*, 139–147. doi:10.1016/j.aquatox.2010.02.003.
42. Vogs, C.; Johanson, G.; Näslund, M.; Wulff, S.; Sjödin, M.; Hellstrandh, M.; Lindberg, J.; Wincent, E. Toxicokinetics of perfluorinated alkyl acids influences their toxic potency in the zebrafish embryo (*Danio rerio*). *ES&T* **2019**, *53*, 3898–3907. https://doi.org/10.1021/acs.est.8b07188.

43. Jantzen, C.E.; Annunziato, K.A.; Bugel, S.M.; Cooper, K.R. PFOS, PFNA, and PFOA sub-lethal exposure to embryonic zebrafish have different toxicity profiles in terms of morphometrics, behavior, and gene expression. *Aq. Tox.* **2016**, *175*, 160–170.
44. Hagenaars, A.; Stinckens, E.; Vergauwen, L.; Bervoets, L.; Knapen, D. PFOS affects posterior swim bladder chamber inflation and swimming performance of zebrafish larvae. *Aqu. Tox.* **2014**, *157*: 225–235.
45. Martínez, R.; Navarro-Martín, L.; Luccarelli, C.; Codina, A.E.; Raldúa, D.; Barata, C.; Tauler, R., Piña, B. Unravelling the mechanisms of PFOS toxicity by combining morphological and transcriptomic analyses in zebrafish embryos. *Sci. Total Environ*. **2019**, *674*, 462–471. https://doi.org/10.1016/j.scitotenv.2019.04.200.
46. Sant, K.E.; Jacobs, H.M.; Borofski, K.A.; Moss, J.B.; Timme-Laragy, A.R. Embryonic exposures to perfluorooctanesulfonic acid (PFOS) disrupt pancreatic organogenesis in the zebrafish, *Danio rerio*. *Environ. Poll.* **2017**, *220*, 807–817.
47. Kherzi, A.; Fraser, T.W.K.; Nourizadeh-Lillabadi, R.; Kamstra, J.H.; Berg, V.; Zimmer, K.E.; and Ropstad, E. A mixture of persistent organic pollutants and perfluorooctanesulfonic acid induces similar behavioural responses, but different gene expression profiles in zebrafish larvae. *Int. J. Mol. Sci.* **2017**, *18*, 291. doi:10.3390/ijms18020291.
48. Annunziato, K.; Doherty, J.; Lee, J.; Clark, J.M., Liang, W.; Clark, C.W.; Nguyen, M.; Roy, M.A., Timme-Laragy, A.R. Chemical characterization of legacy aqueous film-forming foam sample and developmental toxicity in zebrafish (*Danio rerio*). *EHP*, **2020**, 128, 097006-1 to 097006-13. https://doi.org/10.1289/EHP6470.
49. Ding, G.; Zhang, J.; Chen, Y.; Wang, L.; Wang, M.; Xiong, D.; Sun, Y. Combined effects of PFOS and PFOA on zebrafish (*Danio rerio*). *Arch. Environ. Contam. Toxicol.*, **2013**, *64*, 668–675. doi.org/10.1007/s00244-012-9864-2.
